# Operationalizing Integrated Immunization and Family Planning Services in Rural Liberia: Lessons Learned From Evaluating Service Quality and Utilization

**DOI:** 10.9745/GHSP-D-19-00012

**Published:** 2019-09-23

**Authors:** Allyson R. Nelson, Chelsea M. Cooper, Swaliho Kamara, Nyapu D. Taylor, Topian Zikeh, Cefanee Kanneh-Kesselly, Rebecca Fields, Iqbal Hossain, Lolade Oseni, Birhanu S. Getahun, Anne Fiedler, Anne Schuster, Hannah Tappis

**Affiliations:** a Jhpiego, Monrovia, Liberia.; b Jhpiego, Baltimore, MD, USA.; c John Snow, Inc., Monrovia, Liberia.; d John Snow, Inc., Arlington, VA, USA.

## Abstract

**Background:**

Integration of family planning and immunization services provides an opportunity to meet women's need for postpartum family planning and infants' vaccination needs through client-centered care, while reducing financial and opportunity costs for families. The United States Agency for International Development's Maternal and Child Survival Program (MCSP) supported the Liberia Ministry of Health to scale up integrated family planning and immunization services as part of a broader service delivery and health systems recovery program after the Ebola epidemic.

**Methods:**

We conducted a mixed-methods program evaluation in 22 health facilities in Grand Bassa and Lofa counties. Family planning uptake and immunization dropout rates at project sites were compared to rates at 18 matched health facilities in the same counties. We conducted 34 focus group discussions with community members and 43 key informant interviews with health care providers and managers to explore quality of care and contextual factors affecting provision and use of integrated services including postpartum family planning.

**Results:**

From November 2016 to July 2017, 1,066 women accepted referrals from immunization to family planning counseling (10% of all vaccinator-caregiver interactions); the majority of women who were referred (75%) accepted a family planning method the same day. Trends indicated slightly higher family planning uptake in intervention over nonintervention facilities, but differences were not statistically significant. Pentavalent vaccine dropout rates did not increase in intervention compared to nonintervention facilities indicating no negative impact on utilization of immunization services. Clients and providers expressed that the integrated services reduced costs and time for the clients, educated mothers about postpartum family planning, and ensured infants were completing their vaccinations. Providers expressed the need for increased human resources to meet the elevated demand for family planning counseling services and additional focus on community-level social and behavior change activities. Both groups emphasized that social stigma and norms about postpartum sexual abstinence prevented many women from seeking postpartum family planning services.

**Conclusion:**

Although scaling up integrated family planning-immunization services may be programmatically feasible and acceptable to clients and providers, the intervention's success and ability to understand and quantify impact are driven by the effect of contextual factors and fidelity to the intervention approach. Contextual factors need to be understood before implementation, measured during implementation, and addressed throughout implementation to maximize the approach's impact on service utilization and health outcomes.

## Abstract (precis)

Providers, managers, and clients valued the integrated service delivery model. Trends indicated slightly higher family planning uptake in intervention facilities, but that difference was not statistically significant. Intrafacility referrals by postpartum women did not negatively affect immunization utilization rates.

## BACKGROUND

Even before the 2013–2015 Ebola epidemic that severely limited access to and trust in the health system, Liberia's maternal mortality ratio and child mortality rate were among the highest in the world, at 1,072 deaths per 100,000 live births and 94 deaths per 1,000 live births, respectively.^1^ An analysis of data from 21 low- and middle-income countries demonstrated that 61% of all postpartum women have an unmet need for contraception.^2^ In Liberia, one-third of all married women have an unmet need.^1^ Although the overall modern contraceptive prevalence rate increased from 19% in 2013^1^ to 31% in 2016,^3^ these figures still reflect low contraceptive use. Modern postpartum family planning (PPFP) use at 6 months postpartum in Liberia is extremely low at 4%, and an estimated 14% of women of reproductive age are postpartum in a given year.^1^ These statistics highlight opportunities for investments in PPFP use to improve overall modern contraceptive prevalence in the country. Low use of family planning in the period after childbirth results in frequent, closely spaced pregnancies and increased risk of adverse maternal and child health outcomes.^4^^,^^5^ Liberia also has concerning gaps in immunization coverage, especially following the Ebola epidemic. Nationwide coverage of the third dose of the pentavalent vaccine (Penta3) remained low at 68% in 2016^3^ compared to 71% in 2013.^1^

Missed opportunities exist for providing comprehensive health care to caregivers and children during contacts with the health system. The concept of “missed opportunities” resonates for both immunization and family planning sectors alike. The Statement for Collective Action for Postpartum Family Planning^6^ highlights how a “no missed opportunities approach” leverages contacts across the continuum of care through a woman's pregnancy postpartum period to offer family planning counseling and services. For immunization, according to the World Health Organization, a missed opportunity is^7^:

*any contact with health services by an individual (child or person of any age) who is eligible for vaccination (e.g. unvaccinated or partially vaccinated and free of contraindications to vaccination), which does not result in the person receiving one or more of the vaccine doses for which he or she is eligible*.

Each caregiver is expected to seek vaccination services for their child 5 times in the first year of life. These visits present opportunities to discuss and offer health care services beyond immunization, such as family planning. Similarly, family planning counseling and service delivery visits are opportunities for providers to review a client's health card and that of their child to ensure they have received all the necessary care, including vaccines, on schedule. Addressing missed opportunities may enhance efficiency for clients and the health system, provide a more client-centered care approach to service delivery, and contribute to reducing maternal and child mortality.

Integrating routine infant immunization and family planning services has been identified as a promising high-impact practice for family planning.^4^ Prior studies demonstrated a positive effect of family planning and immunization service integration on family planning outcomes.^8^^–^^10^ Evidence of the effect on immunization outcomes is less clear, although at least 2 studies have demonstrated no negative impact.^9^^,^^10^ At the global level, more evidence is needed to understand how integration affects provision and use of family planning and immunization services in different health systems and contexts.

Integrating routine infant immunization and family planning services has been identified as a promising high-impact practice for family planning.

## PROGRAM DESCRIPTION

The United States Agency for International Development (USAID)/Maternal and Child Survival Program (MCSP) worked with the Ministry of Health (MOH) from 2015 to 2018 to restore confidence in the health system following the Ebola epidemic, increasing quality of and demand for maternal and child health services. MCSP expanded an approach to optimize and integrate immunization and family planning contacts to align with the Government of Liberia's *Investment Case for RMNCH for 2016–2020*^11^ commitment to:

*optimize efficiency through improved productivity, and integrating RMNCAH [reproductive, maternal, newborn, child, and adolescent health] service delivery with other vertical programs, while ensuring continuum of care.*

The objective of the approach was to reduce missed opportunities for care in 22 hospitals and clinics in Grand Bassa and Lofa counties.

We built on experience generated by the USAID/Maternal and Child Health Integrated Program (MCHIP) and MOH pilot in 2012, whereby vaccinators were trained to share brief family planning messages and refer postpartum women to same-day co-located family planning services at 10 health facilities in Bong and Lofa counties.^8^ This earlier initiative contributed to impressive increases in the number of new contraceptive users, but the approach did not focus on referrals to immunization from family planning, and the effect of integration on immunization outcomes was less clear. In 2015, when routine service delivery resumed post-Ebola epidemic, MOH endorsed expansion of the approach to additional counties, pending some adjustments to the approach to strengthen and monitor immunization outcomes and ensure the intervention had no negative impact on the likelihood that mothers would return for vaccination services.

Missed opportunities exist for providing comprehensive health care to caregivers and children during contacts with the health system.

MCSP expanded the former MCHIP-MOH integrated approach to include intrafacility referrals for women and their children from the family planning provider to the vaccinator, in addition to the referrals from immunization to family planning. In this expanded model, family planning providers reviewed the child health cards of postpartum women who had come with their infant to the health facility primarily for family planning services, if available, and provided reminders about their child's next vaccination date. If the date had already passed, they referred the woman and child to the vaccinator for same-day immunization services. Vaccinators continued to share family planning messages and refer postpartum women for same-day family planning counseling from the family planning provider, similar to the MCHIP model. They also provided PPFP leaflets to clients who were interested but needed more time to discuss the possibility with their families before deciding to visit the family planning provider. Both vaccinators and family planning providers indicated in their registers if they provided or received a referral. We also introduced a mechanism for tracking referrals to family planning that were completed on a different day, whereby women who did not accept a family planning method were given a special referral card along with a leaflet about the benefits of family planning and asked to return with the card if they decided to come back for family planning services on a different day. Family planning providers used special symbols in their register to track same-day versus different-day referral completion. Under the MCHIP approach, facilities where immunization services were offered in a public space were provided privacy screens because formative inquiry revealed norms discourage return to sex and seeking family planning before the baby walks, resulting in a concern about being seen going for family planning during the extended postpartum period.^8^ Due to logistical constraints, however, privacy screens were only available after the study period presented here. To help ensure privacy during the referral process, the MCSP model called for this referral system to be used only in health facilities and not during immunization campaigns or outreach services in communities.

The MCSP implementation approach was designed to be a scalable version of the MCHIP-MOH approach integrated into existing routine service delivery and supervision structures. In October 2016, MCSP together with MOH conducted a 1-day supervisor training for county- and district-level clinical supervisors and facility officers-in-charge. This training was immediately followed by a 2-day skills-based training for vaccinators and family planning providers from intervention facilities in each county, including a 1-day practical exercise in health facilities that focused on use of the communication tools and techniques and the referral mechanisms. Providers carried job aids, posters, brochures, and referral cards back to their facilities and initiated the integrated services. MCSP and county health team staff conducted on-site mentoring and coaching at each health facility monthly throughout the 9-month study period as part of routine supervision to ensure continuity and quality of the integrated services approach. This approach differed from the MCHIP intervention in which separate project supervision visits were conducted specifically to follow up on family planning–immunization integration.

MCSP included a pragmatic evaluation to glean additional insights into implementation of service integration in a fragile health system and to assess the effect of integration on immunization outcomes. It was designed to examine clients' and health workers' perspectives regarding the quality of care provided with the integrated approach, contextual factors affecting the implementation of the approach, and the effect of service integration on immunization and PPFP service use through the extended postpartum period in intervention facilities in Lofa and Grand Bassa counties. This case study presents qualitative evaluation findings, along with select quantitative findings, to illustrate lessons that can guide design and implementation of future health service integration programs and evaluations.

## METHODS

### Study Design and Facility Selection

We conducted a mixed-methods program evaluation in the 22 health facilities in Grand Bassa and Lofa counties, where the integrated approach was introduced in 2016, and an additional 18 matched, nonintervention facilities in the same counties.

We conducted a mixed-methods program evaluation in health facilities where the integrated approach was introduced in 2016 and in matched, nonintervention facilities.

We compared service utilization at intervention and nonintervention facilities within the constraints of MCSP workplans for the 2 counties. The study design was guided by programmatic opportunities, not calculation of sample sizes and study duration required to detect an effect in family planning or immunization outcomes. In Lofa and Grand Bassa counties, 40 of 47 purposively selected health facilities receiving MCSP support were eligible for integrated immunization-family planning services at the time of study site selection (September 2016); eligibility was defined as having at least 1 vaccinator and at least 1 family planning provider on staff at the facility providing Expanded Programme on Immunization (EPI) and family planning services and recording data from these services in ledgers. Of these facilities, 36 had pair-matches based on the following criteria (in order): county; level of health facility (i.e., hospital, health center, or clinic); type of health facility (i.e., public or private); Penta3 coverage (June to August 2016); and facility catchment area. (If Penta3 coverage was similar for >2 health facilities, selected pairs were based on most similar catchment population.) Paired facilities were assigned to either intervention or comparison arms using a coin toss. We integrated family planning and immunization services in the 18 matched health facilities and the 4 nonmatched health facilities. The majority of facilities were public clinics. Overall, catchment populations were higher in Grand Bassa County facilities compared to Lofa County facilities (Table 1).

**TABLE 1.** Baseline Characteristics of Intervention and Comparison Facilities in Liberia, by County, May to October 2016

Facility Characteristics	Grand Bassa County		Lofa County	
	Intervention Facilities	Comparison Facilities	Intervention Facilities	Comparison Facilities
**Catchment population (2016),**^a^ **No.**	8,280	7,421	3,772	2,901
**Facility type, No.**				
Hospital	0	0	2	2
Health center	0	0	0	0
Clinic	11	11	4	4
**Facility ownership, No.**				
Public	9	9	1	1
Private faith-based	2	2	5	5
**Facility monthly client load** ^b^				
Immunization clients: Pentavalent 3 doses administered, mean (SD)	33.8 (14.5)	32.4 (17.7)	16.9 (15.8)	17.7 (10.0)
Family planning clients: Total family planning users, mean (SD)	119.7 (118.3)	66.2 (80.7)	77.8 (121.2)	81.9 (193.1)

Abbreviation: SD, standard deviation.

a Median facility catchment population among all facilities in the study group.

b Monthly mean number of clients among all facilities in the study group during baseline period: May to October 2016.

### Quantitative Data Collection and Analysis

We used routine health management information systems (HMIS) data to monitor family planning and immunization outcomes at all 36 matched study sites over a 15-month period, including 6 months before intervention and 9 months of program implementation. We intentionally did not introduce a supplementary PPFP data collection register because it would be neither scalable nor sustainable outside of MOH's routine HMIS. Instead, providers made markings in existing registers when referrals were made or completed. We also collected and analyzed monthly data on referrals between the immunization and family planning service points, including the number of referral acceptors (immunization to family planning and family planning to immunization), the proportion of those referrals that were completed on the same or a later date, and the proportion of referral completers who accepted a family planning method on the same day from all intervention facilities.

To assess trends in family planning and immunization service utilization across all health facilities, we reviewed changes in aggregate monthly service delivery statistics before and after integration of family planning and immunization services, disaggregating by county. Primary outcome indicators included number of first and third pentavalent vaccine doses administered at fixed and outreach facilities, pentavalent vaccine dropout rate, and total number of family planning users. Total number of family planning users was defined as the total number of new and continuing modern contraceptive users, excluding vasectomy and condom users, who were excluded for consistency with previous studies^12^ and to isolate the effect of the intervention on the target population. Total family planning users served as the primary family planning indicator rather than new users alone because facilities varied greatly with regard to how the indicator “new family planning users” was understood and captured in HMIS. In this article, descriptive findings are presented with further explanation of methodological limitations to highlight lessons learned regarding challenges in measuring results of integration efforts in real-world settings.

Additionally, we attempted to assess if a statistically significant difference existed in the use of family planning between intervention and comparison facilities. Although we present findings from this analysis in Supplement, we note a poor model fit. This poor fit may be due to a high variation in baseline service utilization levels between facilities, the relatively small sample size dictated by the geographic scope and duration of the project, lack of data available to match facilities on sociodemographic characteristics of facility catchment populations, or other unobserved confounding factors.

MCSP and MOH staff verified both HMIS and referral data quality during routine supervision visits. Outlier data including instances in which the monthly number of family planning users exceeded the total number of women of reproductive age in the catchment population were excluded from analysis along with their paired facility. This resulted in the exclusion of 1 pair of facilities from the family planning analysis. During routine supervision, we discovered notable spillover of the intervention to comparison facilities in Lofa that hindered our ability to detect intervention effects. The intervention spillover occurred in almost all nonintervention facilities in Lofa (6) and none in Grand Bassa, as a result of facility staff and MOH supervisors discussing the approach at quarterly review meetings and carrying the idea from one facility to the next to introduce it outside the scope of our project intervention.

### Qualitative Data Collection and Analysis

To explore perceptions of family planning-immunization service quality and understand how contextual factors affected integration, we conducted 34 focus group discussions (FGDs) (see Table 2) during the ninth month of program implementation in 4 comparison and 12 intervention facilities purposively selected to include low-, average-, and high-performing facilities in each county. For the purpose of qualitative sampling, performance was determined based on the percentage of mothers whose children received vaccination and were referred to family planning and the percentage of mothers who received a family planning method and received referral for same-day immunization services in February to March 2017. Eligible FGD participants were mothers with infants under 1 year of age who attended either family planning or EPI services at an intervention or comparison study site and fathers with infants under 1 year of age in the communities around these health facilities who were prospectively recruited in June 2017. Homogenous FGDs included mothers from intervention facilities who either accepted or did not accept a referral to family planning from EPI or mothers/fathers with children under age 1 in the community. The study team was unable to convene any focus groups with women who accepted referrals to immunization from family planning because these referrals were not as well tracked in practice as the referrals to family planning.

**TABLE 2.** FGD and KII Participants, by County and Intervention Group, Liberia, 2017

	Number of Participants				
	Intervention Facilities	Comparison Facilities	County and National Level	District Level	Total
**Grand Bassa County**					
**FGD**					
Family planning referral acceptors	48	N/A			48
Family planning referral nonacceptors	47	N/A			47
Fathers of <1 children	30	N/A			30
Mothers of <1 children	N/A	19			19
**KII**					
Supervisor/manager	2	0	2	3	7
Family planning provider	5	1			6
Vaccinator	6	2			8
**Lofa County**					
**FGD**					
Family planning referral acceptors	54	N/A			54
Family planning referral nonacceptors	53	N/A			53
Fathers of <1 children	17	N/A			17
Mothers of <1 children	N/A	19			19
**KII**					
Supervisor/manager	2	0	4	2	8
Family planning provider	5	2			7
Vaccinator	6	1			7
National-level manager			1		1
Total	275	44	7	5	331

Abbreviations: FGD, focus group discussions; KII, key informant interviews.

We also conducted 43 key informant interviews (KIIs) with family planning providers, vaccinators, managers, and clinical supervisors. Providers were from sampled health facilities, and managers and supervisors were convenience sampled from participating counties and districts. Providers and supervisors who had assumed their posts within the 3 months prior to recruitment were excluded from the sample due to their limited experience with the integration activities.

All participants were over the age of 18 and provided written informed consent prior to enrollment. Those who were not literate had a witness to the informed consent process. Focus groups lasted on average 75 minutes (max: 120 minutes), and KIIs lasted 45 minutes (max: 90 minutes). All FGDs and KIIs were conducted in a place with visual and auditory privacy. No participants withdrew before the end of the study.

FGDs had a moderator and note-taker; 31 of 34 FGDs consented to be audio recorded. FGD questions were asked in Liberian English and further explained in local dialects (Kissi, Lorma, and Bassa) when necessary. All KIIs were conducted in English and audio recorded.

The qualitative research team included 5 external consultants with experience conducting qualitative research; all participated in a 3-day training and 1-day pilot. The team reviewed and expanded on KII and FGD notes within 48 hours and provided further details after listening to the audio recording. The lead researcher developed an initial coding structure aligned with the research objectives, applied it to all notes, elaborated to incorporate emergent subthemes, and then conducted a second round of analysis. Divergence between participant type and geography were noted. Illustrative quotes representing majority and minority opinions were identified and included in the results where possible. Both rounds of coding and analysis were conducted using Excel.

### Ethical Considerations

Ethical approval and oversight in Liberia was provided by the University of Liberia Institutional Review Board (IRB) (Protocol No. 17-01-022) and in Baltimore, Maryland, United States, by the Johns Hopkins Bloomberg School of Public Health IRB (IRB No. 00007524).

## RESULTS

### Referral Rates at Intervention Facilities

**FIGURE 1:**
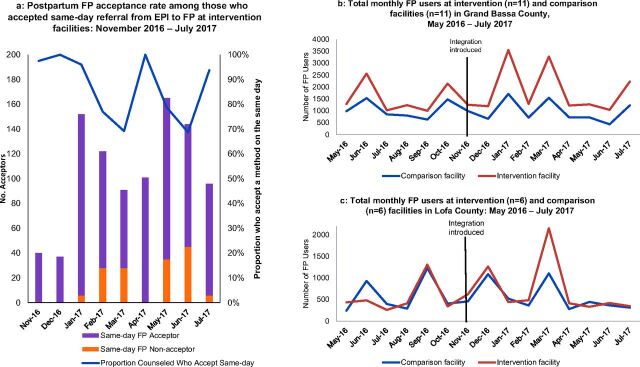
Family Planning Outcomes Abbreviations: EPI, Expanded Programme on Immunization; FP, family planning; PPFP, postpartum family planning.

During the 9-month study period from November 2016 to July 2017, 1,066 women accepted same-day referrals from immunization to family planning in the intervention facilities. Ten percent (1,066/10,519) of all vaccinator-client interactions resulted in a caregiver accepting a same-day referral to family planning. (“Total interactions” is defined as the total number of Penta1 + Penta2 + Penta3 + measles vaccine doses administered at fixed sites, meaning if a child is fully vaccinated, each child-caregiver pair has 4 interactions with the vaccinator after the initial birth-dose vaccinations.) Of those who were referred, 89% (948 of 1,066) completed the referral to the family planning provider on the same day and were counseled by the family planning provider, and 75% (799 of 1,066) accepted a family planning method on the same day. On average, 5.3% of clients (monthly range: 0.7%–11%) who interacted with the vaccinator for their child's vaccines accepted a family planning method on the same day. From month to month during the study period, 70%–100% of women who accepted a referral for family planning counseling accepted a method on the same day (Figure 1a). An additional 164 women (1.6% of 10,519 vaccinator-client interactions) were documented to have completed the family planning referral on a different day.

During the 9-month study period, 1,066 women accepted same-day referrals from immunization to family planning in the intervention facilities.

During the 9-month study period, 456 mothers with infants were referred by a family planning provider to a vaccinator on the same day. The total number of postpartum women eligible for referral was not tracked due to limitations of data available in the routine health information system. Of those who were referred for a same-day vaccination, 71% (323) completed the referral and their child received vaccines on the same day. Referral completion rates were 90% or greater for the majority of facilities that were correctly tracking referrals. Documented referral completion rates were higher in Lofa (94%) than in Grand Bassa (58%). An increasing trend occurred in the number of referrals per month throughout the intervention period (from 15 in November 2016 to 77 in July 2017). Findings from KIIs and FGDs revealed that family planning providers more commonly provided vaccination date reminders rather than same-day referrals to infant immunization because it was uncommon for the child's vaccination date to be on the same day the mother came for family planning counseling.

### Effect of Intervention on Service Utilization

The total number of family planning users at intervention facilities increased during the implementation period in intervention facilities across both counties. Trends indicated slightly increased family planning uptake in intervention facilities over the nonintervention facilities in a comparison of the periods before and after integration (Table 3 and Figure 1b-c). Total family planning users at intervention facilities increased by 19% in February to April 2017 (4–6 months after integration) compared to May to July 2016 (4–6 months before integration) in Grand Bassa, compared to an 11% decrease in total family planning users in comparison facilities in the same period. In Lofa, intervention facilities experienced a 160% increase in total family planning users in a comparison of the same time periods, while the comparison facilities only experienced a 12% increase (Table 3). There did not appear to be a statistically significant difference in likelihood of family planning uptake over these 3 periods in a comparison of nonintervention to intervention facilities (Supplement). Periodic spikes in monthly family planning users corresponded to family planning contraceptive weeks, wherein family planning counseling and services were promoted through community outreach. Quarterly, rather than monthly, comparisons were made to take such spikes into account. Due to a lack of funding, a contraceptive week did not take place in the May to July 2017 quarter (Q5). This prevented our comparison of family planning utilization between Q1 and Q5.

**TABLE 3.** Total Family Planning Users in Intervention and Comparison Facilities, by Quarter and County, Liberia, 2016–2017

County	Pre-intervention		Post-intervention			Comparison		
	Q1 (May–Jul 2016)	Q2 (Aug–Oct 2016)	Q3 (Nov 2016–Jan 2017)	Q4 (Feb–Apr 2017)	Q5 (May–Jul 2017)	% Change (Q3–Q1)	% Change (Q4–Q1)	% Change (Q5–Q1)^a^
**Grand Bassa County**								
Comparison facilities	3,363	2,914	3,365	2,986	2,388	0%	−11%	−29%
Intervention facilities	4,862	4,378	5,998	5,786	4,538	23%	19%	−7%
**Lofa County**								
Comparison facilities	1,566	1,924	2,062	1,749	1,121	32%	12%	−28%
Intervention facilities	1,174	2,059	2,323	3,049	1,098	98%	160%	−6%

a The Ministry of Health conducted Family Planning Contraceptive Week in Q1–Q4, but not in Q5 due to resource constraints.

Trends indicated slightly increased family planning uptake in intervention facilities over nonintervention facilities.

No statistically significant difference was apparent in contraceptive method mix between intervention and comparison facilities, nor between pre- and postintegrated service delivery (data not shown). Across all comparison and intervention facilities, the preferred family planning methods were injectables (61% of women) and oral contraceptives (34%). Note that although providers were encouraged to counsel on lactational amenorrhea method (LAM) as part of the method mix, LAM use was not tracked through the routine health information system and thus LAM users are not included in the analysis.

**FIGURE 2:**
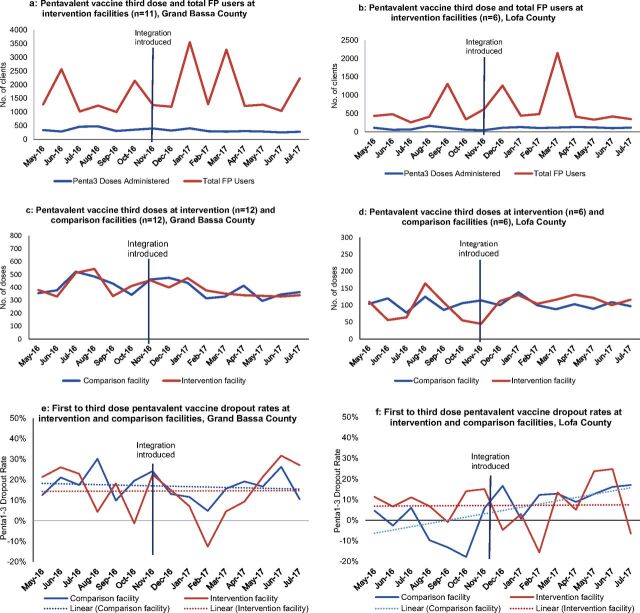
Immunization Outcomes, May 2016-July 2017 Abbreviation: FP, family planning.

The trends in doses administered of first and third dose pentavalent vaccine were similar in intervention and comparison facilities in Lofa and Grand Bassa Counties (Figure 2a-d). In Grand Bassa, a slight decreasing trend occurred in the number of Penta1 and 3 doses administered over the baseline and intervention periods. This decreasing trend was consistent with trends observed in other counties not implementing integrated service delivery.^3^ Quarterly spikes in vaccination rates corresponded to the periodic intensification of routine immunization campaigns carried out by MOH.

In Grand Bassa, the dropout rate was mostly constant in intervention facilities before and after the integrated approach was introduced at approximately 15%; the dropout rate in comparison facilities was consistently higher than in intervention facilities. The dropout rate was also constant in intervention facilities in Lofa at approximately 8% before and after the intervention was introduced; the dropout rate dramatically increased in comparison facilities during the same time period (Figures 2e-f). These results demonstrate no negative impact of the intervention on immunization doses administered or dropout.

### Perceptions of Quality of Services

#### Client Perspectives

Clients generally expressed positive feedback about their experience with the integrated services and with immunization and family planning services more broadly. However, some concerns were noted regarding embarrassment to accept family planning referrals in a public setting, including being seen walking from one service room to the other. Both acceptors and nonacceptors expressed appreciation for the convenience of receiving both services on the same day, for being reminded on an ongoing basis about the availability of both services, and for having an opportunity to learn more about a service other than the one they originally sought.

*Before when we used to come, the vaccinator used to vaccinate our children, but he never used to tell us about family planning. But this time, now as soon as we finish with the vaccine, they can show us [a] poster and tell us about the family planning. That it is good.* —Mother, age 18–19, family planning referral nonacceptor, Lofa County

*I feel happy with [integrated service delivery] because this time when I come I can just use my two cards and get my baby vaccine and family planning. —*Mother, age 35–39, family planning referral acceptor, Lofa County

Most mothers indicated that they felt the service integration should continue. Even several of the family planning referral nonacceptors indicated that they hoped the vaccinator would continue to encourage them about family planning during future visits to potentially change their minds. Minority opinions expressed among a few family planning referral nonacceptors were that mothers had already made up their minds to not use family planning and they did not want to continually have it raised, vaccinators should simply focus on providing immunization services, and other mothers might feel overly pressured to accept a family planning method with it being brought up repeatedly.

Most mothers indicated that they felt the service integration should continue.

The main reasons cited by family planning referral nonacceptors for not following through on referral from immunization to family planning services were concerns about partner opposition to use of family planning and about being seen in a public setting agreeing to meet with the family planning provider. The latter concern was particularly noted where immunization services and family planning information are provided in a group format or where a client must walk through the waiting area to get to the family planning services area.

*The reasons why some agree to come on different day for family planning may be because at first the person who was telling them about family planning was telling them among the group of people, so they were ashamed and decided to come on different day. —*Mother, age 20–24, family planning referral nonacceptor, Grand Bassa County

*The family planning will be good for you, but when I tell my man, he [is] always saying no. Every time he say[s], “We will go so you can get it,” but to no avail. —*Mother, age 25–29, family planning referral nonacceptor, Lofa County

When asked whether other mothers might feel reluctant to return for immunization if the vaccinator talks about family planning, most respondents said there was no concern and they liked that the vaccinator explained the benefits of birth spacing and encouraged them to visit the family planning provider. However, a few referral nonacceptors mentioned that women might feel embarrassed or upset because they do not want to talk about family planning due to the perceived negative health effects. One mother mentioned,

*Some will feel bad because when they bring their children for vaccine[s], family planning will be discussed, and some don't want to take family planning. —*Mother, age 35–39, family planning referral nonacceptor, Grand Bassa

Other family planning referral nonacceptors felt that women would not be discouraged but would instead be pleased to hear about family planning; for example:

*Other women will feel good because they are telling them about family planning and at the same time vaccinating their children. —*Mother, age 20–24, family planning referral nonacceptor, Lofa County

For referrals from family planning to immunization, mothers widely mentioned that the family planning provider had checked their child's vaccination card and reminded them when to return or referred them immediately if their child was overdue for a vaccine. Mothers welcomed this support from the family planning provider to remind them when to come back for their child's vaccines.

Mothers welcomed the provider reminding them when to return for their child's vaccines.

Mothers were asked their overall perspectives on the immunization and family planning services at the health facilities. Most mothers had a positive impression of immunization services, with some commenting that vaccines are regularly available, that services are free of charge, that mothers appreciate receiving regular reminders about when to return for the next vaccine, and that they appreciated the way the vaccinator spoke to them. For example:

*We can feel good. The vaccine man can smile with us, give us seat, and also explain the importance to us about the vaccine. —*Mother, age 35–39, family planning referral nonacceptor, Grand Bassa County

Several mothers also mentioned gaps in immunization services, including long waiting time and lack of availability of certificates for immunization schedule completion and vaccination cards.

Regarding family planning services, mothers (including the family planning referral nonacceptors) felt largely positive about the services. They appreciated the way the family planning provider discussed the subject, that services are free of charge, and that providers allowed women to make their own choices on which method to use. A couple of mothers mentioned areas that need to be addressed, including improvements in privacy, better explanations of family planning side effects by family planning providers, improvements in the family planning providers' interpersonal skills, and prevention of stock-outs of contraceptives.

*As for me where they are giving the family planning, the curtain should be closed because it is short, and when people are passing to go to the vaccine place while the family planning woman is talking to you, they can see and hear you. —*Mother, age 30–34, family planning referral acceptor, Lofa County

Mothers felt positive about the services, but noted privacy, provider communication, and education could be improved.

Recommendations suggested by mothers for service improvements include having more health education and behavior change efforts at the community level, addressing privacy concerns, and increasing space for provision of immunization services.

#### Service Provider Perspectives

Vaccinators and family planning providers expressed largely positive views about the integrated approach. Service providers mentioned broad benefits for clients as well as a number of benefits of the service integration for the service providers themselves, including helping them locate “lost” mothers and children for each service and enabling them to reach more clients for both services. One vaccinator mentioned that “it has made mothers to be more free with us,” and another expressed that he appreciated learning more about family planning and assisting with family planning service provision. Several health workers said they saw improvements in recordkeeping systems, with increased focus on using the data to make decisions at the facility.

Providers said a benefit of service integration was the ability to reach more clients for both services.

Vaccinators and family planning providers widely described having positive working relationships with each other, with many indicating that the relationships had improved with the intervention. Service providers generally indicated that they coordinate to ensure that mothers receive both services, compare registers on a regular basis, and support each other to ensure both roles are covered if one provider is called offsite.

*The new approach has affected our interaction positively, in that we both compare all of our EPI [Immunization]-family planning record[s] on a daily basis. —*Vaccinator, Grand Bassa County

As noted by a program manager in Lofa:

*… the EPI and the family planning providers are closer now as compare to before in terms of relationship…I think because they both receive the same training to provide the services together. They are now working together. —*Program Manager, Lofa County

Vaccinators, in particular, saw great value from the family planning and immunization integration training, largely around improving their understanding of family planning and enabling them to play a role in family planning service provision. For example:

*The major change is that I never knew young baby mothers could take family planning and I did not think mothers were going to accept, but now the young mothers are accepting beyond what I expected. —*Vaccinator, Lofa County

The main implementation challenges mentioned by health workers included perceived increased workload, documentation challenges to keep track of referred clients, resistance to family planning use among some clients, and commodity stock-outs.

Several supervisors and service providers indicated that the service integration had increased their work responsibilities and time required to provide services. One vaccinator mentioned now spending more time on each client, and a nurse discussed the challenge of juggling antenatal care, family planning, immunization, and other clients, which resulted in increased wait time and dissatisfaction among some clients. For example, one nurse from Lofa noted:

*Many of the patients complain on the waiting time because we can leave our big belly [antenatal care clients] and other patients to take care of the family planning and EPI clients.*

Respondents revealed that the heavy workload occasionally led to “services not being fully implemented” and to gaps in the recordkeeping.

Overall, respondents indicated that in spite of the workload challenges, they had come to see integrated family planning–immunization services as part of their job functions. One supervisor noted, for example:

*Well, they [vaccinators and family planning providers] are saying that the new approach … is very good and that many clients are coming for those services as compared to before, but … they complain a lot on workload. They are saying the work is too much, but at the end of the day I make them to understand that it is [in] our duties.* —Supervisor, Grand Bassa County

Despite the challenges, all vaccinators and family planning providers indicated that they would like to see the integrated approach continue. Two vaccinators mentioned that future efforts should engage community health workers to increase engagement on PPFP and immunization at the community level. Others said the approach should be scaled up to additional facilities and counties.

All vaccinators and family planning providers indicated that they would like the integrated approach to continue.

### Contextual Factors Affecting Integrated Service Delivery Outcomes

Contextual factors affecting service integration outcomes included social stigma, misconceptions and concerns about negative health effects of family planning, human and material resource availability, and organization of services within the facility. Mothers and fathers expressed misconceptions regarding the health effects of family planning methods, including on their own health, the quality of breastmilk, and the health of their child. Stigma and fear of social judgment for using PPFP, especially while mothers have small babies (i.e., during a period of socially prescribed postpartum abstinence), were repeatedly raised as a concern across the client respondent groups. Several respondents indicated that they had not known prior to the intervention that “family planning is good for young baby mothers.” Several mothers discussed how use of family planning was seen as a violation of a traditional norm for women to wait to resume sexual activity until after the baby walks or the child is weaned, as illustrated here:

*Some people will say in the community you want [to] do plenty man business [sex], so you do not want to wait for your child to walk*. —Family planning referral nonacceptor, age 30–34, Grand Bassa County

The view that postpartum women are seen in a negative light if they use PPFP pervaded the FGDs, especially among the family planning referral nonacceptors. Along with this pressure to not be seen using family planning before the baby walks, women indicated that they also feel pressure from their husbands to return to sexual activity:

*They will say it is because of man business, and we, the Muslim[s], we have 40 days after you deliver, and after that you [are] forced to have sex with your husband, and I don't want my man to go to different woman. —*Family planning referral nonacceptor, age 25–29, Lofa County

*Men will always want to have sex with women who have young children, [but] the breastmilk will spoil and the child will not walk [early]. —*Family planning referral nonacceptor, age 20–24, Lofa County

FGD participants said that postpartum women are seen in a negative light if they use PPFP.

Most fathers expressed a positive general impression about family planning, although many were hesitant to endorse *postpartum* family planning. Fathers expressed concerns that family planning might lead to side effects, that it might facilitate infidelity, or that they may incur judgment from others in the community for using family planning soon after childbirth:

*Because the baby is still small, the shame will come on the man.*” —Father, age 35–39, Grand Bassa County

Most fathers also indicated that they feel comfortable speaking with their wives about family planning. Fathers widely recognized the importance of immunization services for their children.

When asked how they felt about women receiving both family planning information and services while bringing children for vaccination at the health facility, the vast majority of husbands participating in the focus groups were supportive. They described benefits such as convenience to the mother (“It saves time”), increased knowledge for the mother that she can share with the father, and the reinforcement of information from both service areas as an additional motivator:

*It also helps women to educate husbands who are not knowledgeable about family planning. —*Father, age 30–34, Lofa County

*For me, I feel happy because they are coming and getting two services at the same time. —*Father, age 20–24, Grand Bassa County

Only 1 husband expressed reservations about the service integration:

*I am not happy with the idea because some of our mothers and sisters are not educated. So, therefore, they will not be able to catch the idea at the same time. —*Father, age 25–29, Lofa County

Respondents indicated that provision of integrated services was affected by human and material resources as well as the facility setup. Specific factors that they identified included commodity availability, staff attrition, and availability of separate rooms for provision of each service.

Several respondents noted the need for refresher training and for orienting new staff due to staff turnover.

*Some of those that were trained to implement the integration services have left some of the facilities…The new ones are not able to do the work properly, and they also do not have that much interest in it because they did not get the training. We still need periodic training to get the new staff involved. —*Program Manager/Supervisor, Lofa County

Supervisors recognized the need for additional staff, and one reported having advocated for more staff, but “the only reply you can get is, ‘no money.’”

Service providers and supervisors expressed mixed experiences with whether or not stock-outs had posed a challenge. Overall, respondents had no major stock-out concerns, but a few noted occasional shortages of family planning commodities; for example:

*There is always shortage in the commodities before the month ends. —*Facility Supervisor, Grand Bassa County

At least 1 facility supervisor indicated increasing commodity projections due to increased demand for vaccines and family planning commodities as a result of the intervention. Several respondents also indicated that the facility setup did not allow for sufficient privacy, affecting client uptake of referrals from one service to the other because clients did not want to be seen walking from the immunization area to the family planning area. They suggested having both services in the same private place; for example:

*There is no specific place for this service. —*Program Manager/Supervisor, Lofa County

*One thing that will help to improve this service is to have all of the resources, logistics, [in a] private room for clients. —*Facility Supervisor, Grand Bassa County

Several respondents indicated that the facility setup did not allow for sufficient privacy.

## DISCUSSION

Our study's qualitative results demonstrate that both providers and clients view the integration of family planning and immunization services as a positive development that reduces the time and cost for families seeking health services, improves coordination and implementation of services at health facilities, and promotes client-centered care. Although we were not able to demonstrate statistically significant changes in overall family planning outcomes, our findings demonstrate no negative impact on immunization dropout rates. Given the lack of clear findings from MCHIP on the impact of the intervention on immunization dropout rates, this study provides reassurance that service integration does not negatively affect immunization service utilization and uptake. Our results reinforce that integration of family planning and immunization services has the potential to be a promising strategy for encouraging postpartum contraceptive uptake. Even in a postemergency setting with a shortage of health workers, where social norms stigmatize the practice of PPFP and immunization coverage is relatively low, integrated referrals were positively received and did not adversely affect immunization outcomes, indicating using the reciprocal referral approach provides added value.

Integrating family planning and immunization services has the potential to encourage postpartum contraceptive uptake, without affecting use of immunization services.

A few factors limited our ability to detect statistically significant changes in overall family planning outcomes, including (1) the use of “total family planning users” as the primary outcome of interest (rather than “new family planning users” which would be more sensitive in capturing postpartum use), (2) not tracking LAM users in the number of total family planning users, (3) the small sample size as a result of small health facility catchment areas and client loads, and possibly, (4) the reduced intensity of supervision specifically for this intervention through this scaled approach compared to the MCHIP approach. LAM use had contributed to a substantial portion of the family planning method mix among immunization-referred clients within the MCHIP work.^8^ Fluctuations in service utilization due to family planning and immunization campaigns and seasonality affecting accessibility during heavy rains also affected our ability to detect significant changes in family planning utilization data. Prior studies of efforts to integrate family planning and immunization services in Togo, Nepal, and Rwanda demonstrated positive results on family planning outcomes with no negative effects on immunization outcomes.^9^^,^^10^^,^^13^

To our knowledge, this article is the first immunization and family planning integration publication to document the results and challenges of bidirectional integration with referrals to immunization as well as to family planning. While the cumulative numbers of clients with same-day referrals from family planning to immunization were relatively small, the more frequent appointment reminders through the family planning platform may have contributed to complete and timely immunizations, although timeliness should be further explored.

### Design and Implementation Considerations of Future Integration Efforts

Questions remain regarding the most effective approaches for expanding and sustaining the effectiveness of service integration efforts within resource-constrained environments. Challenges with maintaining continuity and fidelity of family planning and immunization service integration implementation to intervention design were noted by studies in Rwanda and Ghana.^12^ Many of the contextual factors affecting service integration through this expanded approach in Liberia, such as stigma and privacy concerns, were consistent with those identified in the MCHIP study.^8^

Questions remain regarding the most effective approaches for expanding and sustaining the effectiveness of service integration efforts within resource-constrained environments.

Many women accepted referrals from immunization to family planning and received the information about PPFP positively. For other women, however, lack of privacy within the health facility was a major barrier to accept and follow through on referrals to family planning. Health workers noted the importance of continued education and engagement on the topic of PPFP to address misconceptions. Although family planning–immunization integration is an avenue to reduce missed opportunities for family planning uptake, addressing social stigma and norms through community-based activities could further improve PPFP uptake. Uptake could also be improved by resolving privacy concerns at the facility, including altering patient flow and protecting privacy during both immunization and family planning consultations. Additional programming with a focus on both provider and community behavior change and community engagement would complement the facility-level integration of services.

Providers also noted the benefits of increased team collaboration, although human resource shortages often affected providers' ability to expedite family planning clients referred from immunization. At the facility level, a team dynamic was evident at many of the intervention facilities. The family planning provider and vaccinator were perceived as “married”—they met regularly to discuss clients' use of postpartum services and how to increase referrals and ensure quality services were being provided and recorded. This teamwork should be emphasized in future programs because it improved the quality of services, particularly the vaccinator's family planning counseling and strategies to improve referral follow-through, and contributed to the sustainability of integrated efforts. Notably, although providers perceived increased workload as a result of the intervention, referred clients represented a small proportion of all clients and the data do not reflect dramatic increases in service uptake. The perceptions of added workload more likely reflect the effort of coordination with the other service providers and additional tracking requirements, as well as shifts in workflow, rather than increases in workload due to an increased demand for services.

Future programmatic efforts in Liberia should incorporate reflection on findings from the intensive MCHIP effort and MCSP's more scalable model. From these experiences, the most essential inputs and activities that we believe should be included within future expanded implementation efforts are summarized in the Box.

BOX **Key Inputs and Activities for Success of Family Planning and Immunization Service Integration**

- Conducive policy environment and strong engagement at national and subnational levels
- Formative assessment to inform program strategy
- Training for service providers and orientations for supervisors which includes values clarification, increasing knowledge of postpartum family planning (PPFP) and addressing misconceptions, and practical skills application
- Routine supportive supervision (as part of existing supervision mechanisms) with attention to improving quality of integrated service delivery and data trends
- Dedicated space for immunization and family planning service provision, including addressing privacy concerns through altered patient flow, privacy screens, or other means
- Availability and use of communication materials for clients and job aids for providers
- Tracking and regularly monitoring immunization and PPFP outcomes (including lactational amenorrhea method) at facility and subnational levels
- Community-level activities to increase knowledge and acceptance of PPFP

The integrated approach can be scaled once a cadre of trained clinical supervisors exists and resources exist for a brief, on-site training and the few inputs described in the Box. We have demonstrated this approach is possible even in the context of a fragile health system. Once the integration is successfully operating at scale, we would suggest minimal intrafacility referral tracking in order to minimize the burden on health workers. Importantly, implementers should further explore opportunities to align facility service integration with community engagement to address barriers to uptake, namely social norms around postpartum abstinence.

### Considerations for Monitoring and Evaluation of Integrated Services

To rigorously assess the effect of the intervention on uptake of family planning among women bringing their children for routine immunization services, it is necessary to specifically monitor trends in PPFP and, if possible, to monitor LAM use as part of the family planning method mix. Due to our focus on implementing a scalable intervention, we chose to rely on HMIS indicators. As PPFP acceptors and LAM users were not captured in the routine HMIS, we were unable to monitor these potentially very valuable data. The addition of PPFP-specific indicators to the HMIS is critical to effective monitoring of the effect of interventions on PPFP at scale. In addition, promoting use of PPFP, especially in contexts with entrenched stigma around its use, is a long-term behavior change intervention. Additionally, longer observation time periods may be necessary to detect these shifts in PPFP perceptions and intentions and the resultant impact on PPFP uptake. The full effect of the intervention is neither realized nor reported by capturing same-day referral data only because the effects of the intervention may be indirect and at times delayed. For example, conversations within families and communities (such as those sparked by the leaflets) may result in other women in the community deciding to seek PPFP, even if they were not the ones who originally interacted with the vaccinator. On the other hand, some women may also choose to seek PPFP services long after the day they received PPFP information from the vaccinator and therefore may not be captured as a referral acceptor if they do not mention their interaction with the vaccinator as their reason for seeking services.

Assessing how the intervention affects contraceptive uptake among women bringing their children for routine vaccination requires monitoring PPFP trends.

The extent of the intervention spillover to comparison facilities in Lofa County demonstrated buy-in and interest from the county health teams and the scalability of the approach because the intervention was organically carried to new facilities by supervisors and staff moving between different facilities. However, it resulted in a diluted effect of the intervention between intervention and comparison facilities. Future initiatives should consider study design variations that maximize internal and external validity.^14^ Factors to consider include appropriate data source selection, client caseloads in the study area, and adequate study duration to detect statistically significant changes in service utilization, as well as the conditions required for more advanced multilevel analyses measuring contamination and spillover effects.^15^

A recent commentary exploring lessons to date regarding integration of health services noted the complexities of these efforts and highlighted that the wide variation in how services are implemented, depending on the context, poses challenges in “rigorously evaluating” these initiatives.^16^ Future efforts should focus on application of “complexity aware” measurement approaches, which align monitoring and evaluation of program interventions with implementation realities. As noted by the CORE Group in its Complexity Matters Call to Action^17^:

*But while outcome evaluation may usefully generate hypotheses, it generally reveals little about the process of change. In the real-world of implementation, controlling for context is not possible. Retrospectively knowing “what worked” in a particular program… does not reliably answer the question of “what works” in general and what will work in future programs.*

Our understanding of family planning–immunization service integration could benefit from future studies that consider interdisciplinary approaches that allow for more exploration of context,^18^ the “how” of implementation,^19^ intervention costs, and the drivers of social and behavior change. These approaches must also elucidate and model incremental changes that may underlie routine service data trends over longer time horizons.^20^ Future studies could examine communities' and clients' exposure to the intervention and their knowledge, intention, and motivation for seeking PPFP and immunization services.

Future studies could explore factors such as service integration context and the drivers of social and behavior change.

## CONCLUSION

Overall, this intervention capitalizes on existing resources to improve the quality of services for women and children and minimize missed opportunities to improve health outcomes. This service integration approach was focused on process improvement and was neither resource intensive nor difficult to implement. This study demonstrates that although scaling up integrated family planning-immunization services may be programmatically feasible and acceptable to clients and providers, the success of the intervention and ability to understand and quantify impact are driven by the effect of contextual factors and fidelity to the intervention approach. Contextual factors need to be understood to the greatest extent possible before implementation, measured through complexity-aware measurement approaches during implementation, and addressed throughout program implementation to maximize the impact of the approach on service utilization and health outcomes.

This study contributes to learning on the “how” of family planning and immunization service integration, that is, the local contextualization of an integrated service delivery model, the requirements for program inputs and implementation, fidelity to intervention design and adaptations that occur in the real world, a larger-scale approach, and the influence of health systems and social factors on the adaptation and effectiveness of the integrated approach. Understanding all these context-specific factors describing the “how” is critical as we consider the future of family planning-immunization integration as a “promising” high-impact practice for promotion of PPFP. Now that this integration approach has been adopted by the Government of Liberia and incorporated into the Liberia Family Planning Costed Implementation Plan: 2018–2022, our ambition is for the government and other stakeholders to apply and build on this learning within programs at scale, with attention to the essential elements we have highlighted, as part of a toolbox of approaches to further address gaps in PPFP uptake and promote more holistic care for women and children.

## Supplementary Material

19-00012-Nelson-Supplement.pdf

## Notes

**Peer Reviewed**
