# Supplementary material for: Operationalizing Integrated Immunization and Family Planning Services in Rural Liberia: Lessons Learned From Evaluating Service Quality and Utilization
Source: Glob Health Sci Pract. 2019 Sep 23;7(3):418–34. doi: 10.9745/GHSP-D-19-00012 (PMC6816810; doi:10.9745/GHSP-D-19-00012)
Supplement: 19-00012-Nelson-Supplement.pdf [file 19-00012-Nelson-Supplement.pdf]

SUPPLEMENT TABLE 1. Likelihood of Use of Family Planning Services Among Intervention and Comparison Health Facilities in Lofa and Grand Bassa Counties, Liberia May 2016 to July 2017

**Indicator**	**Likelihood of Family Planning Use (Incidence Risk Ratio)[a]**	**95% CI (SE)**
Intervention group	1.45	0.793, 2.642 (0.444)
Immediate intervention effect (November 2016-January 2017)	1.09	0.920, 1.299 (0.096)
Long-term intervention effect (February-July 2017)	0.90	0.738, 1.103 (0.093)

Abbreviations: CI, confidence interval; SE, standard error.

[a]Generalized linear model with a negative binomial distribution and robust variance adjusted for time, facility level (hospital or clinic), and county (Lofa or Grand Bassa).
